# Supplementary material for: Prevalence and factors associated with moderate-to-severe anaemia among virally suppressed people with HIV at a tertiary hospital in Zambia
Source: PLoS One. 2024 Aug 26;19(8):e0303734. doi: 10.1371/journal.pone.0303734 (PMC11346727; doi:10.1371/journal.pone.0303734)
Supplement: Attachment — Submitted filename: Response to Reviewers.docx [file pone.0303734.s004.docx]

We appreciate the valuable feedback and additonal comments provided by you and the reviewers on our manuscript titled “**Prevalence and Factors Associated with Moderate-to-Severe Anaemia Among Virally Suppressed People with HIV at a Tertiary Hospital in Zambia**” (PONE-D-24-17294). We have carefully considered each comment and have made the necessary revisions to address them. See comment responses below

Reviewer comment no.1

Thank you so the authors for looking at the linear association with outcome comment; however, it does not appear that they assessed linearity of association. Usually, the variable would be categorized in quartiles and then run in a bivariate model for the outcome with a reference category and the betas then plotted to assesses whether the categorical estimates fall on roughly a straight line. I think, but I am not certain, the authors may have assessed the normality of the distribution. It surprises me that all of the continuous variables would demonstrate a linear association.

Our response

**Thank you for your valuable feedback on our manuscript. Initially, we assessed the linearity of the logit using the Box-Tidwell transformation. It is a robust method for testing the linearity assumption in logistic regression models with continuous independent variables . This technique involves creating interaction terms between each continuous variable and its natural logarithm, allowing us to evaluate if the logit (log-odds) of the outcome is linearly related to the independent variables. If the interaction term is statistically significant, it indicates a departure from linearity which was not the case for these variables. To further address your concerns, we have categorized each continuous variable into quartiles, conducted bivariate logistic regression, and plotted the beta coefficients against the quartile midpoints. This plot demonstrated that the beta coefficients fell on a roughly straight line, indicating a linear relationship. We hope that this reinforce the validity of our findings and address your concerns regarding the linearity of the associations.**

**Binary logistic regression results after categorising into quartiles**

**Age**

| Variables in the Equation | | |  |  |  |  |  |  |  |
| --- | --- | --- | --- | --- | --- | --- | --- | --- | --- |
|  |  | B | S.E. | Wald | df | Sig. | Exp(B) | 95% C.I.for EXP(B) | |
|  |  |  |  |  |  |  |  | Lower | Upper |
| Step 1a | Agegroupquartiles | |  | 8.31 | 3 | 0.04 |  |  |  |
|  | Agegroupquartiles(1) | 0.597 | 0.335 | 3.162 | 1 | 0.075 | 1.816 | 0.941 | 3.505 |
|  | Agegroupquartiles(2) | 0.808 | 0.323 | 6.271 | 1 | 0.012 | 2.243 | 1.192 | 4.221 |
|  | Agegroupquartiles(3) | 0.889 | 0.327 | 7.374 | 1 | 0.007 | 2.434 | 1.281 | 4.624 |
|  | Constant | -2.428 | 0.269 | 81.241 | 1 | 0 | 0.088 |  |  |
| a Variable(s) entered on step 1: Agegroupquartiles. | | | | | |  |  |  |  |
|  |  |  |  |  |  |  |  |  |  |

**BMI**

| Variables in the Equation | | |  |  |  |  |  |  |  |
| --- | --- | --- | --- | --- | --- | --- | --- | --- | --- |
|  | `1 | B | S.E. | Wald | df | Sig. | Exp(B) | 95% C.I.for EXP(B) | |
|  |  |  |  |  |  |  |  | Lower | Upper |
| Step 1a | bmigroupedquartiles | | | 3.608 | 3 | 0.307 |  |  |  |
|  | bmigroupedquartiles(1) | -0.513 | 0.288 | 3.17 | 1 | 0.075 | 0.599 | 0.34 | 1.053 |
|  | bmigroupedquartiles(2) | -0.236 | 0.278 | 0.723 | 1 | 0.395 | 0.79 | 0.458 | 1.361 |
|  | bmigroupedquartiles(3) | -0.072 | 0.267 | 0.073 | 1 | 0.787 | 0.93 | 0.551 | 1.571 |
|  | Constant | -1.604 | 0.185 | 74.941 | 1 | 0 | 0.201 |  |  |
| a Variable(s) entered on step 1: bmigroupedquartiles. | | | | | |  |  |  |  |

**CD4 count**

| Variables in the Equation | | |  |  |  |  |  |  |  |
| --- | --- | --- | --- | --- | --- | --- | --- | --- | --- |
|  |  | B | S.E. | Wald | df | Sig. | Exp(B) | 95% C.I.for EXP(B) | |
|  |  |  |  |  |  |  |  | Lower | Upper |
| Step 1a | CD4countgroupedquartiles | | | 1.303 | 3 | 0.728 |  |  |  |
|  | CD4countgroupedquartiles(1) | 0.08 | 0.272 | 0.086 | 1 | 0.769 | 1.083 | 0.635 | 1.847 |
|  | CD4countgroupedquartiles(2) | -0.166 | 0.284 | 0.344 | 1 | 0.558 | 0.847 | 0.485 | 1.477 |
|  | CD4countgroupedquartiles(3) | -0.193 | 0.287 | 0.454 | 1 | 0.501 | 0.824 | 0.47 | 1.446 |
|  | Constant | -1.725 | 0.195 | 78.301 | 1 | 0 | 0.178 |  |  |
| a Variable(s) entered on step 1: CD4countgroupedquartiles. | | | | | |  |  |  |  |
|  |  |  |  |  |  |  |  |  |  |

**Lymphocytes**

| Variables in the Equation | | |  |  |  |  |  |  |  |
| --- | --- | --- | --- | --- | --- | --- | --- | --- | --- |
|  |  | B | S.E. | Wald | df | Sig. | Exp(B) | 95% C.I.for EXP(B) | |
|  |  |  |  |  |  |  |  | Lower | Upper |
| Step 1a | Lymphogroupedquartiles | | | 11.923 | 3 | 0.008 |  |  |  |
|  | lymphogroupedquartiles(1) | 0.404 | 0.324 | 1.552 | 1 | 0.213 | 1.498 | 0.793 | 2.827 |
|  | lymphogroupedquartiles(2) | -0.517 | 0.378 | 1.865 | 1 | 0.172 | 0.597 | 0.284 | 1.252 |
|  | lymphogroupedquartiles(3) | -0.797 | 0.407 | 3.827 | 1 | 0.05 | 0.451 | 0.203 | 1.002 |
|  | Constant | -1.619 | 0.239 | 45.938 | 1 | 0 | 0.198 |  |  |
| a Variable(s) entered on step 1: lymphogroupedquartiles. | | | | | |  |  |  |  |
|  |  |  |  |  |  |  |  |  |  |

Reviewer comment 2

Thank you to the authors for including missing data as a limitation; however, I don't think it received as in depth an interrogation as needed. If the data is not missing at random or completely at random, the inferences may not be valid at all. A bit more discussion about why this level of information bias is unlikely to affect the inferences from the observed associations is warranted with that level of missing data.

Our response

**Thank you for your feedback regarding the limitation of missing data in our study. We acknowledge the need for a more in-depth discussion. Our analysis showed that missing data were due to laboratory analysis challenges and incomplete patient information in the smart care system. We determined that the missing data were Missing Completely at Random (MCAR), meaning the probability of missingness was unrelated to any observed or unobserved data. We appreciate the opportunity to further enhance our manuscript.**

**See added more detailed discussion of missing data in this study (see lines 318 to 322)**
